# Application of multi-method-multi-model inference to radiation related solid cancer excess risks models for astronaut risk assessment

**DOI:** 10.1016/j.zemedi.2023.06.003

**Published:** 2023-07-08

**Authors:** Luana Hafner, Linda Walsh

**Affiliations:** a Swiss Federal Nuclear Safety Inspectorate ENSI, Industriestrasse 19, 5201 Brugg, Switzerland; b Department of Physics, University of Zürich, Winterthurerstrasse 190, 8057 Zürich, Switzerland

**Keywords:** Radiation Attributed Decrease of Survival, Multi-Model Inference, Solid Cancer Risks, Astronaut Risk Assessment, Epidemiology

## Abstract

The impact of including model-averaged excess radiation risks (ER) into a measure of radiation attributed decrease of survival (RADS) for the outcome all solid cancer incidence and the impact on the uncertainties is demonstrated. It is shown that RADS applying weighted model averaged ER based on AIC weights result in smaller risk estimates with narrower 95% CI than RADS using ER based on BIC weights. Further a multi-method-multi-model inference approach is introduced that allows calculating one general RADS estimate providing a weighted average risk estimate for a lunar and a Mars mission. For males the general RADS estimate is found to be 0.42% (95% CI: 0.38%; 0.45%) and for females 0.67% (95% CI: 0.59%; 0.75%) for a lunar mission and 2.45% (95% CI: 2.23%; 2.67%) for males and 3.91% (95% CI: 3.44%; 4.39%) for females for a Mars mission considering an age at exposure of 40 years and an attained age of 65 years. It is recommended to include these types of uncertainties and to include model-averaged excess risks in astronaut risk assessment.

## 1 Introduction

Methodologies currently applied by the various national space agencies for radiation protection of astronauts during and after space missions have already been reviewed in this special issue (see paper from TG115 group [1]). Radiation protection of astronauts currently involves detailed radiation risk assessment by some agencies involving the application of radiation related cumulative risk measures. For example NASA [2], applies the Risk of Exposure Induced Cancer or Death (REIC or REID) (Vaeth and Pierce [3]). Recent developments, not yet adopted for operational use by national agencies, relate to the quantity Radiation Attributed Decrease of Survival (RADS) [4], [5]. RADS has been pointed out to provide more accurate risk estimates for astronauts, because in this calculation method, fewer types of population specific rates from the general population are integrated, where such rates represent a major source of uncertainty (Walsh et al. [6]). Generally, it is important to assess uncertainties and to optimize their magnitude if possible to generate reliable risk assessments. Several research groups have pointed out the importance of uncertainty assessment in cancer risk calculations for astronauts and have considered different types of uncertainties. Hafner et al. [7] and Walsh et al. [6] have considered the uncertainties of the risk per unit dose, risk effect modifiers, EAR risk scaling, neutron RBE and of the baseline rates. However, there are even more sources of uncertainties that could be considered in the calculation process. One example is the uncertainty involved in model choice which can be accounted for by implementing model-averaged excess risks. Stabilini et al. [8] have used Multi-Model Inference (MMI) to calculate eight model averaged excess risks based on the all solid cancer incidence models currently published and deemed plausible by the scientific community. In the present study, the model averaged excess risks from Stabilini et al. [8] have been used to calculate the radiation attributed decrease of survival (RADS) in order to discuss the impact of the uncertainty of model choice on the space radiation related all solid cancer incidence risk assessment. Previous work by Stabilini et al. [8] applied several methods to investigate the impact on the MMI results of different approaches for modelling the baseline cancer rates in the individual models contributing to MMI. This was done because the risk models based on stratified baselines, which have many parameters for categories of attained age and other covariables, are penalised much more strongly in the calculation of the MMI model weights than the models based on parametric baselines. Based on these multi-method considerations, a new Multi-Method-Multi-Model Inference (M^4^I) approach is introduced to provide single RADS estimates for lunar and Mars missions.

## 2 Material and methods

### 2.1 General assumptions

Cumulative risk assessment methods in space radiation research are currently based on radiation risk models derived from the A-bomb Life Span Study (LSS) data. Ideally, space radiation risk models could be modelled directly from data on astronauts that were exposed to ionizing radiation, but because of the low number of subjects, only very poor statistical significance can be achieved. For more reliable risk models, i.e., with a higher statistical significance, radiation risks are modelled based on data of large epidemiological cohorts that bring the advantage of the cohort size and of a long follow-up period. In the present study, the all solid cancer incidence data, used to fit the risk models from Grant et al. [9] based on weighted colon dose, were used, where the weighted colon dose is generally used to estimate all solid cancer risks in the LSS studies. The A-bomb survivors in the LSS data were exposed to gamma and neutron radiation. To calculate the total equivalent organ dose, the neutron contribution to the total equivalent organ dose is weighted with a Relative Biological Effectiveness (RBE) of 10. An RBE of 10 has been used in the LSS studies (e.g. Grant et al. [9]), however recent studies have shown that the neutron RBE may be higher than 10 (e.g. Cordova and Cullings [10]) and the impact of higher neutron RBEs on radiation related cancer incidence risk has been discussed (Hafner et al. [11]). This neutron RBE is therefore applied to obtain the risk models from the LSS data and needs to be distinguished from the space neutron RBE, appropriate for the neutron fluence of galactic cosmic radiation and solar radiation. As Walsh et al. [6] state, the LSS neutron RBE is not directly applicable to astronaut dosimetric monitoring data as a proxy for neutron space RBE. Ideally, the risks based on the unit weighted LSS organ dose should be applied to the unit organ dose equivalents calculated from actual astronaut dosimetry monitoring data and appropriate space RBEs from different space radiation types (i.e., radiation quality factors) taken from authoritative assessment (e.g. [12]). In the future these weights or equivalent organ doses from the space radiation needs to be applied in space radiation risk assessments. However, this is out of the scope of the present study. In extended assessments, quality factors used to calculate organ dose equivalents from the space radiation need to be applied in space radiation risk assessments. However, this is out of the scope of the present study which aims to show the impact of model uncertainty (see however the paper by Walsh et al. [13] this issue, which shows the impact of the choice of two different sets of radiation quality factors on the organ dose equivalent estimates).

### 2.2 Calculation of RADS

The calculation of RADS based on all solid cancer incidence has been performed analogously to the method described in Walsh et al. [6].

Therefore, the details of the analysis can be found in Walsh et al. [6] and in the present study only the most directly relevant formulas are denoted.

RADS is a cumulative radiation risk based on the radiation-attributed hazard, H. The radiation-attributed hazard H represents the total integrated excess incidence risks for all solid cancer incidence, and is conditional on survival until a certain age a:

(1) $$RADS\left(a|e,D,RBE\right)=1-exp\left(-H\left(a|e,D,RBE\right)\right)$$

where D is the colon weighted dose, e age at exposure and RBE is the LSS neutron relative biological effectiveness. The hazard is then described as an integral from age at exposure plus some latency period, l, up to any required attained age:

(2) $$H\left(a|e,D,RBE\right)=\int_{e+l}^{a}h\left(u,e,D,RBE\right)du$$

where

(3) $$h\left(a,e,D,RBE\right)=\frac{0.5*ERR\left(D,a,e,RBE\right)m\left(a\right)+0.5*EAR\left(D,a,e,RBE\right)/10000}{\mathrm{DDREF}}$$

is the excess risk that accounts for population age and sex-specific cancer incidence rates m(a) and for a dose and dose rate effectiveness factor (DDREF). ERR is the excess relative risk and EAR the excess absolute risk. The weighting of 0.5 was applied in the present study according to the ICRP (2007) [14] recommendations for all solid cancer risk calculation. ICRP [14] provided no information about the magnitude of the uncertainty of these weighting factors, which are therefore treated as fixed values in the present study. Further, the calculations were performed with an LSS neutron RBE value of 10 in the weighted dose, a DDREF of 2 and average population age and sex-specific cancer incidence rates from Belgium, Denmark, France, Germany, Italy, The Netherlands, Spain and United Kingdom.

In order to account for the uncertainty of model choice in the RADS calculation, the model averaged excess risk estimates for all solid cancer incidence risks from Stabilini et al. [8] were used for the ERR and EAR in Eq. (3). Stabilini et al. [8] have fitted currently known all solid incidence models to the most recent publicly available Life Span Study (LSS) data on the survivors of the A-bombs over Hiroshima and Nagasaki (Grant et al. [9]) and used the Akaike Information Criterion (AIC) and the Bayesian Information Criterion (BIC) as metrics to objectively quantify the goodness of fit. Further, the models were fitted once with the baseline of the given models (which were either stratified or parametric) and once with the best fitting parametric baseline of all models. This procedure was done to compensate for the fact that the models with stratified baselines were strongly penalized in MMI by the AIC or BIC weights due to the large number of baseline strata, which all counted as model parameters. This procedure results in four different model average excess risks for ERR and EAR which are listed in Table 1, along with the mathematical forms of the ER model parts. The full forms of the radiation risk models as well as all values of the fit parameters including the corresponding uncertainties that are used in the present study can be found in Stabilini et al. [8] and the mathematical forms of the ERs are given in Table 1.

**Table 1:** Risk models and MMI weights based on AIC and BIC for the model-averaged excess risk estimates with original and same baseline (BL) from Stabilini et al. [8]. *D* denotes weighted colon dose in Gy, *e* represents age at exposure and *a* the attained age, *s* is a categorical variable for the sex and K the total shielded kerma. Greek letters indicate the fit parameters, i.e., the risk coefficients in the excess risk forms where the same mathematical forms were used for ERR and EAR, but with different fit parameters. AIC – Akaike Information Criterion; BIC – Bayesian Information Criterion; EAR – excess absolute risk; ERR – excess relative risk; MMI – multi-model inference

Model	Excess risks	MMI ER, AIC weights				MMI ER, BIC weights			
		Original BL		Same BL		Original BL		Same BL	
		EAR	ERR	EAR	ERR	EAR	ERR	EAR	ERR
*BEIR VII phase 2*[17]	$\;\alpha_{s}D\exp\left(\tau e^{*}\right){\left(\frac{a}{70}\right)}^{\nu }{\;\;\;e}^{*}=\left\{\begin{array}{c} \begin{array}{cc} \frac{e-30}{10} & e\;<\;30\;years \end{array}\\ \begin{array}{cc} 0 & e\;\geq \;30\;years \end{array} \end{array}\right)$			0.67	0.65			1	0.95
*Grant et al. L*[9]	$\left(\alpha D\right)\exp\left(\tau \left(\frac{e-30}{10}\right)+\nu \ln\left(\frac{a}{70}\right)+\varphi \left(K>4\right)\right)\left(1+\sigma \;s\right)$			0.09	0.08	0.97	0.99		
*Grant et al. LQ*[9]	$\left(\alpha D+\beta D^{2}\right)\exp\left(\tau \left(\frac{e-30}{10}\right)+\nu \ln\left(\frac{a}{70}\right)+\varphi \left(K>4\right)\right)\left(1+\sigma \;s\right)$			0.24	0.18	0.02	0.01		
*Preston et al. L*[18]	$\left(\alpha D\right)\exp\left(\tau \left(\frac{e-30}{10}\right)+\nu \ln\left(\frac{a}{70}\right)+\varphi \left(K>4\right)\right)\left(1+\sigma \;s\right)$					0.01			
*INWORKS-Leuraud L*[19]	$\;\left(\alpha D\right)\exp\left(\tau \left(\frac{e-30}{10}\right)+\nu \left(\frac{a-70}{10}\right)\right)\left(1+\sigma s\right)\;\;\nu =\left\{\begin{array}{cc} \nu_{1} & \;a\;<\;60\;years\\ \nu_{2} & 60\;\leq \;a\;<\;80\;years\\ \nu_{3} & a\;\geq \;80\;years \end{array}\right)$				0.01				
*INWORKS-Leuraud LQ*[19]	$\;\left(\alpha D+\beta D^{2}\right)\exp\left(\tau \left(\frac{e-30}{10}\right)+\nu \left(\frac{a-70}{10}\right)\right)\left(1+\sigma s\right)\;\;\nu =\left\{\begin{array}{cc} \nu_{1} & \;a\;<\;60\;years\\ \nu_{2} & 60\;\leq \;a\;<\;80\;years\\ \nu_{3} & a\;\geq \;80\;years \end{array}\right)$				0.02				
*UNSCEAR L*[20]	$\;\left(\alpha D\right)\exp\left(\sigma \;s+\varepsilon \ln\left(\frac{a-e}{40}\right)+\nu \ln\left(\frac{a}{70}\right)\right)$	0.73	0.73		0.04				0.05
*UNSCEAR LQ*[20]	$\;\left(\alpha D+\beta D^{2}\right)\exp\left(\sigma \;s+\varepsilon \ln\left(\frac{a-e}{40}\right)+\nu \ln\left(\frac{a}{70}\right)\right)$	0.27	0.27		0.02				

To compare the results from RADS calculated with model averaged excess risk estimates with RADS calculated with only one model, RADS was also calculated using the ER based on the linear Grant model from Grant et al. [9]. The impact of MMI is demonstrated with examples here for which RADS is calculated for two missions with hypothetical doses: A 180 days lunar mission (0.17 Sv) and a Mars mission (1 Sv). These hypothetical doses were derived from NASA estimates for Moon and Mars missions at solar minimum and with a 5 g cm^-2^ aluminium shielding of the space craft (NASA [15], Cucinotta [16]). For Mars missions, NASA found hypothetical doses of 1.03 Sv and 1.07 Sv, for simplification a dose of 1 Sv was chosen for Mars missions in the present study.

The 95% CI of RADS were calculated by a Monte Carlo simulation with 1000 realisations. The uncertainties on the ERR and EAR risk coefficients in the calculation of RADS were simulated by using the values of the fit parameters and sampling new fit parameters based on the covariance matrices provided. This means that for each model, attained age, age at exposure, sex and dose value, realisations of the excess risk estimator were generated by randomly sampling the values for all the risk coefficients estimators (defining this excess risk) from their relative asymptotic joint normal distributions (i.e., accounting for their estimated covariance matrices) [8]. Further, the uncertainties of the baseline incidence rates were Poisson sampled and included in the Monte Carlo simulation.

As described above, Stabilini et al. [8] introduced different methods to calculate a model average excess risk, resulting in four different average risk models. Since one general risk estimate would be preferable and there is no evidence to suggest that any one single method can be clearly favoured over the other methods, a multi-method-multi-model inference (M^4^I) approach is introduced in the present study. In this M^4^I-approach, the RADS estimates resulting from the four methods described above, and given in Table 1, were combined to provide one general RADS estimate by taking the weighted mean with weights based on AIC and BIC weights in Table 2. The M^4^I-approach represents a new method of applying an MMI approach to several MMI-results in order to get one general risk estimate. The uncertainties of these general risk estimates were assessed using Gaussian error propagation.

**Table 2:** RADS incidence for different missions calculated with excess risks fitted with the same and original baseline using the model weights based on AIC and BIC. Further, RADS calculated with the Grant et al. [9] model is shown. RADS is calculated at age at exposure 40 and attained age 65. AIC – Akaike Information Criterion; BIC – Bayesian Information Criterion; RADS – Radiation Attributed Decrease of Survival; ER – Excess Risk

RADS calculated with ER based on fit to the original baseline (95% CI) [%]				
	**Lunar mission (0.17 Sv)**		**Mars mission (1 Sv)**	
	Males	Females	Males	Females
BIC model weights	0.46 (0.37;0.59)	0.74 (0.61;0.89)	2.69 (2.13;3.38)	4.27 (3.55;5.08)
AIC model weights	0.37 (0.30;0.45)	0.55 (0.46;0.68)	2.14 (1.73;2.69)	3.21 (2.67;3.96)
Grant model	0.46 (0.37,0.60)	0.74 (0.61;0.89)	2.69 (2.12;3.39)	4.27 (3.54;5.10)

RADS calculated with ER based on fit to the same baseline (95% CI) [%]				

	**Lunar mission (0.17 Sv)**		**Mars mission (1 Sv)**	
	Males	Females	Males	Females

BIC model weights	0.43 (0.34;0.52)	0.72 (0.61;0.84)	2.52 (2.02; 3.01)	4.16 (3.53;4.89)
AIC model weights	0.41 (0.35;0.48)	0.67 (0.60;0.76)	2.45 (2.09;2.84)	4.01 (3.57;4.56)
Grant model	0.42 (0.33;0.52)	0.67 (0.54;0.82)	2.44 (1.89;3.03)	3.90 (3.15;4.75)

## 3 Results

In Table 2, results for RADS solid cancer incidence at an attained age of 65 years for a lunar and a Mars mission is shown for males and females exposed at 40 years of age. Results, with 95% CIs given in parentheses are based on using the three different approaches (MMI based on BIC and AIC weights and the Grant model) to calculate the ER fitted to original and same baselines.

RADS calculated using the original baseline with the model-averaged ER based on BIC weights and RADS calculated with the Grant models result in the same mean estimates for both mission types. The differences in range values of the 95% CIs for all missions only vary between 0.01% and 0.03%. This is expected, because the Grant models contribute the most to the model-averaged ER using BIC weights. For a Mars mission RADS for males is 2.69% (95%CI: 2.13%; 3.38%) using BIC model weights in the ER, 2.14% (1.73%; 2.69%) using AIC model weights in the ER and 2.69% (2.12%; 3.39%) using the linear Grant models in the ER. For females the corresponding estimates are 4.27% (3.55%; 5.08%), 3.21% (2.67%; 3.96%) and 4.27% (3.54%; 5.10%) for ER with BIC weights, AIC weights and Grant models respectively.

RADS estimates calculated with the model-averaged ER based on AIC weights are lower compared to the RADS estimates calculated with the other two methods. The mean estimates in Table 2 differ by between 0.09% and 1.06%.

Considering the ER fitted with the same baseline, RADS for a Mars mission results in 2.52% (2.02%;3.01%) using BIC model weights in the ER, 2.45% (2.09%; 2.84%) using AIC model weights in the ER and 2.44% (1.89%; 3.03%) using the linear Grant models in the ER for males. For females the corresponding RADS estimates are 4.16% (3.53%; 4.89%), 4.01% (3.57%; 4.56%) and 3.90% (3.15%; 4.75%). Generally, the calculation results in very similar RADS estimates for the ER using AIC model weights and the ER based on Grant models, varying in the mean estimate only by values between 0% and 0.11%. RADS calculated with the ER using BIC model weights result in the higher mean estimates than using just the Grant model with differences varying by 0.01–0.26%.

**Figure 1:**
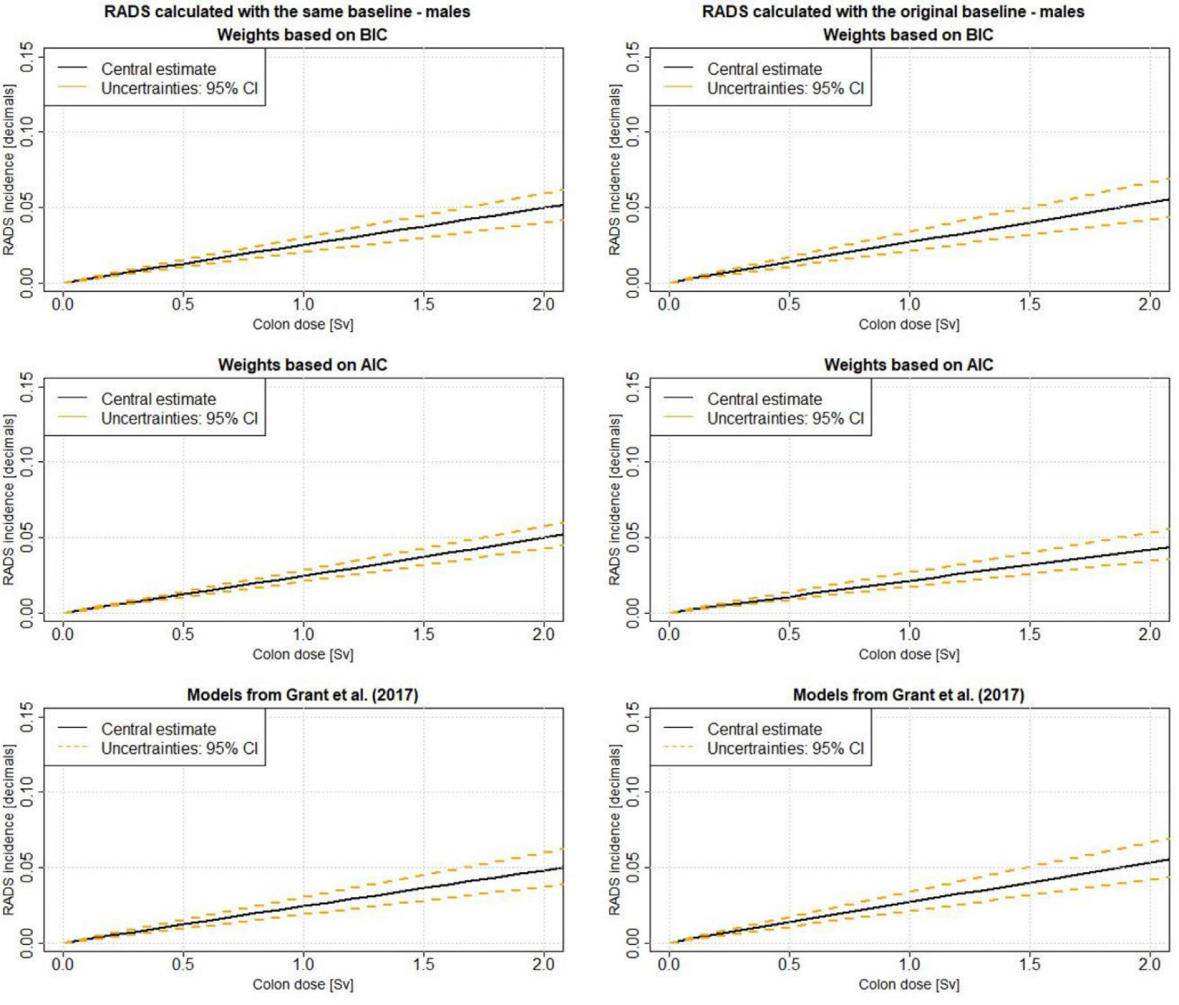
**RADS as function of dose for males:** RADS incidence for males as function of dose calculated with excess risks fitted with the same and original baseline using the model weights based on AIC and BIC. Further, RADS calculated with the Grant et al. [9] model is shown. RADS is calculated at age at exposure 40 and attained age 65. AIC – Akaike Information Criterion; BIC – Bayesian Information Criterion; RADS – Radiation Attributed Decrease of Survival

**Figure 2:**
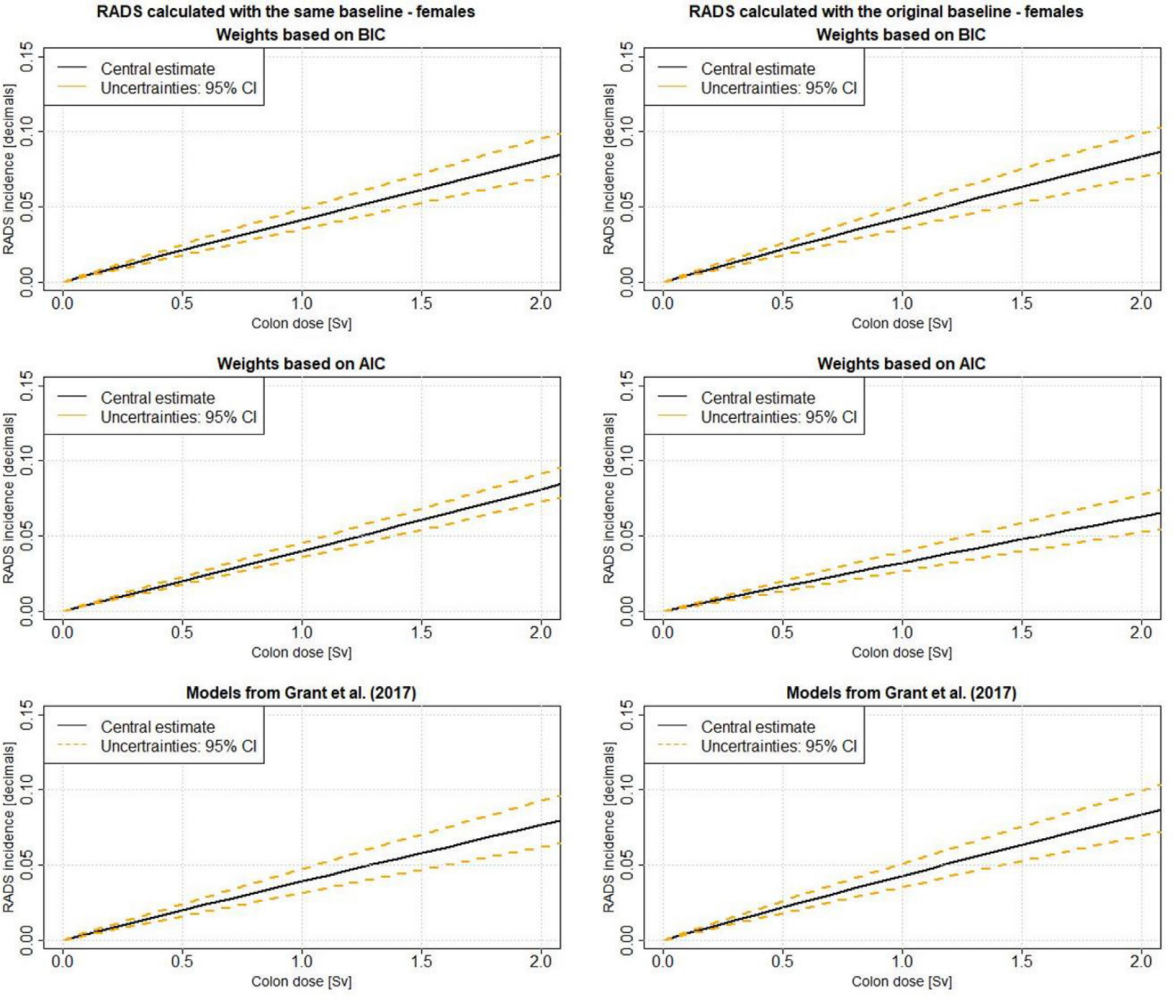
**RADS as function of dose for females:** RADS incidence for females as function of dose calculated with excess risks fitted with the same and original baseline using the model weights based on AIC and BIC. Further, RADS calculated with the Grant et al. [9] model is shown. RADS is calculated at age at exposure 40 and attained age 65. AIC – Akaike Information Criterion; BIC – Bayesian Information Criterion; RADS – Radiation Attributed Decrease of Survival

For both types of baseline fits it can be observed that the 95% CI from RADS using the AIC weights are smaller than the 95% CI of RADS calculated with the other two methods. Fig. 1 demonstrates RADS for males as function of dose calculated with the excess risks fitted once with the same and once with the original baseline using the model weights based on AIC and BIC as well as the linear Grant model. The corresponding results for females are shown in Fig. 2.

In order to get one general RADS estimate, the M^4^I-approach was applied to the RADS estimates based on AIC and BIC weights in Table 2 (excluding the estimates from the Grant model). The general RADS estimates are found to be 0.42% (0.38%; 0.45%) for males and 0.67% (0.59%; 0.75%) for females for a lunar mission and 2.45% (2.23%; 2.67%) for males and 3.91% (3.44%; 4.39%) for females for a Mars mission considering an age at exposure of 40 years and an attained age of 65 years.

## 4 Discussion

Lately more attention has been paid to the uncertainties of different parameters in the radiation related cancer risk calculations (e.g. Ulanowski et al. [4], Hafner et al.[7], Walsh et al. [6], Stabilini et al. [8]) and sensitivity analysis have been performed to analyse their impacts (e.g. Zhang et al. [21]). This issue is not only of concern in space radiation related cancer risk calculations, but for radiation related cancer risk calculations in general radiation protection settings. However, this discussion should be and is a major interest in the research field of space radiation related cancer risk calculation, because of the quantity of additional parameters in the calculation method, which all represent possible sources of uncertainties. A reliable and reasoned risk assessment is needed for high quality decision making when it comes to health risks and possible detrimental health effects after radiation exposure on space missions for astronauts.

In the present study the impact of including model-averaged excess risks into the RADS calculation has been analysed. It can be observed from Table 2, Figure 1, Figure 2 that RADS using ER based on AIC weights result in smaller risk estimates with narrower 95% CI than RADS using ER based on BIC weights. Whether AIC or BIC weights should be used for the purpose of model averaging has been discussed by Stabilini et al. [8]. The researchers have pointed out that AIC tends to favour models which have more parameter than the true models (Kass and Raftery [22]) and that AIC is dimensionally inconsistent (Kashyap [23]). BIC does not have an information-theoretic justification, but in contrast to AIC, BIC is dimensionally consistent and excludes more complex models with higher number of parameters earlier (Claeskens and Hjort [24]). Walsh [25] points out that from theoretical consideration of dimensional consistency BIC appears to be the best method for model selection. Burnham and Anderson [26] discuss the use of AIC and BIC for model selection and conclude that both criteria can be derived as either frequentist or Bayesian procedures, so the choice cannot be argued by being Bayesian or non. If AIC or BIC should be used, must be based on comparing measures of their performance under conditions realistic of applications [26].

RADS calculated using the original baseline with the model-averaged ER based on BIC weights and RADS calculated with the Grant models result in the same mean estimates for all missions, only for females on Mars swing-by mission a variation of 0.01% in the mean can be observed. The range of the 95% CIs for all missions vary between 0.01% and 0.04%. Using the same baseline, the variation for the RADS estimates using ER based on BIC model weights and the ER based on Grant models is larger. The mean estimates as well as the range of the 95% CI vary between 0.01% and 0.27% for the missions considered in the present study.

Summarizing, it can be stated that for a reliable cancer risk assessment, uncertainties in the model choice should be included into the calculation and the total impact is likely to be greater for leukaemia than for the solid cancer outcome investigated here. Additionally, RADS estimates using the ER based on BIC model weights generally result in the highest risk estimates, which assures that the space radiation related cancer risks are not underestimated. Alternatively, also one general RADS estimate can be calculated by applying the M^4^I-approach as shown in the present study. This approach has the advantage of considering all the possible calculation methods for MMI and therefore accounting for another uncertainty of MMI method choice. However, it should be noted that this approach may result in complex risk models compared to when only one model-average method or even just one model is implemented.

Stabilini et al. [8] fitted their models with a centering of the risk effect modifiers around age at exposure 30 years and attained age 70 years, to calculate the standardized excess risks of the general population. Risk estimates for astronauts use generally standardized age of exposure 40 years and attained age 65 years (although NASA are proposing to take the most radiation sensitive case of a 35-year-old female astronaut, then find out which effective dose corresponds to a mean REID of 3%, then apply this effective dose (∼600 mSv) as a limit to all astronauts – a plan not completely supported [27]). Therefore, the fit parameters and models reported by Stabilini et al. [8] are not standardized in their age centering for astronaut risk assessment. Therefore, it is important to pay attention to this age centering when communicating standardized risk estimates. Although age centering is really a calibration for non-linear models to avoid computational difficulties in the Poisson regression numerical algorithm, the judicious choice of centering can represent “average ages of astronauts”, enabling the relevant non cumulative excess risks to be calculated with uncertainties directly as risk model estimates that have direct relevance to astronaut missions. Ideally information on dose, age at exposure and attained age is provided with risk estimates.

A further point that will affect the space radiation risk estimates and the according uncertainty assessment is the use of unit weighted organ dose calculated from actual astronaut dosimetry monitoring data. Because space radiation is composed of different space radiation types and not only of gamma and neutron radiation, the composition of the unit weighted organ dose will be different than for the A-bomb radiation. Since the influence of the RBE of space radiation on the risk estimates for radiation related cancer is still under investigation (e.g., Walsh et al. [13]), the RBE will be major source of uncertainty that needs to be accounted for in future space radiation risk assessments.

Uncertainties are treated differently among the different research groups and a general understanding in the radiation protection community of how uncertainties in radiation risk assessment should be treated is still missing. It is therefore important that the international committees place more emphasis on this subject and provide guidance on how best to deal with uncertainty assessments. For reliable risk assessment, it is necessary to know which uncertainties should be included directly into the calculations, which ones should at least be mentioned in the context and which ones do not necessarily need to be included.

Although not currently used in operational radiation protection of astronauts, the usefulness of the RADS metric has been shown in the previous work cited here, and an extension of this previous work to include MMI has been demonstrated here for the first time. A recent National Academies of Sciences, Engineering, and Medicine report [27] has recommended that metrics other than REID should be re-examined. This report also emphasised the importance of communicating a comprehensive picture of an individual astronaut’s cancer risks due to radiation exposure. Both of these recommendations are in line with the work presented here.

In extended assessments, quality factors used to calculate organ dose equivalents from the space radiation need to be applied in space radiation risk assessments. However, this is out of the scope of the present study which aims to show the impact of model uncertainty (see however the paper by Walsh et al. [13] this issue, which shows the impact of the choice of two different sets of radiation quality factors on the organ dose equivalent estimates). So ideally future space radiation risk assessments will be based on organ dose equivalents and will include MMI in order to provide the most reliable risk estimates possible.

## 5 Conclusion

The usefulness of the RADS metric has been shown in the previous work cited here, and an extension of this previous work to include MMI has been demonstrated here for the first time in line with recent recommendations [27]. The impact of including model-averaged excess risks into the RADS calculation has been demonstrated here and it was shown that RADS applying ER based on AIC weights result in smaller risk estimates with narrower 95% CI than RADS using ER based on BIC weights. Further the M^4^I-approach has been introduced that allows calculating one general RADS estimate providing a weighted average risk estimate. Including uncertainties and model-averaged excess risks in astronaut risk assessment is recommended here.

## Declaration of Competing Interest

The authors declare that they have no known competing financial interests or personal relationships that could have appeared to influence the work reported in this paper.
